# Reducing suicides in mental healthcare: results from a 4-year follow-up implementation study in the Netherlands (SUPRANET)

**DOI:** 10.3389/fpsyt.2024.1080235

**Published:** 2024-04-19

**Authors:** Kim Setkowski, Anton J. L. M. van Balkom, Adriaan W. Hoogendoorn, Gerdien Franx, Marjolein Veerbeek, Remco F. P. de Winter, Renske Gilissen

**Affiliations:** ^1^ Research Department, 113 Suicide Prevention, Amsterdam, Netherlands; ^2^ Amsterdam University Medical Center (UMC), location Vrije Universiteit Amsterdam, Department of Psychiatry, Amsterdam Public Health Research Institute and GGZ inGeest (Mental Health Institution), Amsterdam, Netherlands; ^3^ Department of Psychiatry, Amsterdam Public Health Institute, Amsterdam University Medical Center (UMC), location Vrije Universiteit Amsterdam, Amsterdam, Netherlands; ^4^ GGZ Rivierduinen (Mental Health Institution), Leiden, Netherlands; ^5^ Parnassia Psychiatric Institute, The Hague, Netherlands; ^6^ The School for Mental Health and Neuroscience (MHeNs) Maastricht University, Maastricht, Netherlands

**Keywords:** suicide prevention, implementation, guideline recommendations, benchmarking, mental healthcare, quality of care, national action network, multicentric study

## Abstract

**Objective:**

In 2016, the SUicide PRevention Action NETwork (SUPRANET) was launched. The SUPRANET intervention aims at better implementing the suicide prevention guideline. An implementation study was developed to evaluate the impact of SUPRANET over time on three outcomes: 1) suicides, 2) registration of suicide attempts, and 3) professionals’ knowledge and adherence to the guideline.

**Methods:**

This study included 13 institutions, and used an uncontrolled longitudinal prospective design, collecting biannual data on a 2-level structure (institutional and team level). Suicides and suicide attempts were extracted from data systems. Professionals’ knowledge and adherence were measured using a self-report questionnaire. A three-step interrupted time series analysis (ITSA) was performed for the first two outcomes. Step 1 assessed whether institutions executed the SUPRANET intervention as intended. Step 2 examined if institutions complied with the four guideline recommendations. Based on steps 1 and 2, institutions were classified as below or above average and after that, included as moderators in step 3 to examine the effect of SUPRANET over time compared to the baseline. The third outcome was analyzed with a longitudinal multilevel regression analysis, and tested for moderation.

**Results:**

After institutions were labeled based on their efforts and investments made (below average vs above average), we found no statistically significant difference in suicides (standardized mortality ratio) between the two groups relative to the baseline. Institutions labeled as above average did register significantly more suicide attempts directly after the start of the intervention (78.8 per 100,000 patients, *p*<0.001, 95%CI=(51.3 per 100,000, 106.4 per 100,000)), and as the study progressed, they continued to report a significantly greater improvement in the number of registered attempts compared with institutions assigned as below average (8.7 per 100,000 patients per half year, *p*=0.004, 95%CI=(3.3 per 100,000, 14.1 per 100,000)). Professionals working at institutions that invested more in the SUPRANET activities adhered significantly better to the guideline over time (b=1.39, 95%CI=(0.12,2.65), *p*=0.032).

**Conclusion:**

Institutions labeled as above average registered significantly more suicide attempts and also better adhered to the guideline compared with institutions that had performed less well. Although no convincing intervention effect on suicides was found within the study period, we do think that this network is potentially able to reduce suicides. Continuous investments and fully implementing as many guideline recommendations as possible are essential to achieve the biggest drop in suicides.

## 1 Introduction

In the Netherlands, about 40% of all people who die by suicide were under the care of mental health institutions (1). Guideline recommendations (e.g., continuity of care, ward safety, safety plans, implementation of policies, and staff training) have a significant effect on patient outcomes (2, 3) and may reduce the risk for patients to die by suicide (4). However, it is widely known that in many countries, the guideline recommendations for the treatment of patients with suicidal thoughts and behaviors are still not fully disseminated in clinical practice (5). For example, a study by Teismann et al. (5) investigated clinicians’ knowledge of the German guideline recommendations for the treatment of suicidally depressed patients. They found large variations in clinicians’ compliance with these guideline recommendations. Even though some recommendations were fully adhered to by clinicians, other recommendations were not. For example, only 13.2% of all clinicians indicated that they would offer follow-up contact after a patient had been discharged from inpatient care. This is undesirable, given the fact that immediate post-discharge is a time of marked suicide risk (6). The authors therefore stated that further dissemination is very much needed.

In the Netherlands, the uptake of the suicide prevention guideline in Dutch mental healthcare is also insufficient (7), leading to a gap between recommended evidence-based treatments and their use in clinical practice (8, 9). A number of theoretical frameworks have illustrated that guideline implementation can be complex, with barriers at many levels that may prevent the everyday use of guidelines. For example, a model by Grol & Wensing (10) suggests that the following three criteria should be met to integrate a guideline successfully: 1] the organization of care within every institution needs to be adapted to enable professionals to deliver care by the guidelines (organizational level), 2] professionals must have the knowledge and capacity to execute the guideline recommendations in clinical practice (professional level), and 3] patients must accept the care they receive following the guideline recommendations (patient level).

Some years ago, a one-day training in the Netherlands was developed and evaluated in a cluster randomized trial to promote the implementation of the multidisciplinary guideline (11) among mental health professionals (12). This study found positive results on professionals' competencies and attitudes toward the guideline recommendations (13). However, until now, most Dutch clinicians have still not participated in this training.

To our knowledge, there have been a few studies examining the effectiveness of guideline recommendations on suicide rates within large mental health services. A UK study published in 2012 (14) found the greatest drop in suicide rates for institutions implementing 7 to 9 guideline recommendations simultaneously, compared with institutions implementing fewer recommendations. Three individual service changes led to a significant reduction in suicide risk: 24-hour crisis care, dual diagnosis policy, and multidisciplinary review after suicide. The reduction in suicides was clinically important, with around 200-300 fewer suicides per year (14). In a later UK study, Kapur et al. (4) concluded that organizational factors are important too. They found that organizational factors interacted with individual service changes, suggesting that service changes in organizationally healthy institutions (e.g., showing low rates of staff turnover) seemed to have a larger impact on reduced suicides than institutions with organizational problems (4). Another initiative called the ‘Perfect Depression Care’ program of the Henry Ford Health System in the US was developed to reduce the number of suicides among 200,000 patients within a large network of two hospitals (inpatient) and ten clinics. This was done by 1) assessing every patient for suicidal thoughts and behaviors and 2) performing tailored improvement activities in four domains (partnership with patients, clinical practice, access to care, and electronic data systems) (15). Several indicators (e.g., safety, effectiveness on suicides, and patient satisfaction) were developed to monitor improvement changes over time, using electronic data systems and surveys as data sources (15, 16). Compared to the general US population, they found a convincing reduction in average suicide rates of 96 suicides per 100,000 (1999–2000) to an average of 24 per 100,000 individuals from 2001 to 2010 (16–18). This is a significant reduction of approximately 75 percent. These UK and US studies were the first ones to identify effective strategies for preventing suicides within clinical practice.

In 2016, 13 large Dutch mental healthcare institutions decided to join forces within SUPRANET (SUicide PRevention Action NETwork), sharing a mutual goal to enhance suicide prevention and optimize the quality of care for patients with suicidal thoughts and behaviors (19). This initiative aims to better implement the recommendations from the suicide prevention guideline (11) (continuity of care, safety plans, waiting list duration, and involvement of families/significant others) in order to significantly reduce the number of suicides and suicide attempts in mental healthcare. SUPRANET focuses on institutions in Dutch specialist mental healthcare, given that these institutions play an important role in detecting and treating patients with suicidal urges, but also have a core responsibility to help patients in a suicidal crisis who visit an institution’s outreach psychiatric department (20). Nowadays, a total of 16 Dutch institutions participate in this network, providing care to more than 300,000 patients, of whom 358 died by suicide in 2020.

This study aimed to evaluate the impact of SUPRANET over time compared to the baseline on the following three outcomes: reduced suicide rates, increased registration of suicide attempts, and improved professionals’ knowledge, attitudes, and adherence to the guideline. Suicide attempts have a much higher incidence rate compared with institutions suicides, and even though suicide attempts are not very sensitive in adequately predicting later suicide (21), they are still one of the biggest known risk factors. Currently, suicide attempts are systematically underreported in Dutch clinical practice. Therefore, we consider an improvement in the registration of suicide attempts over time as a positive sign (i.e., indication that patients with suicidality are being signalled on time and that the data is being accurately registered).

We hypothesized that institutions wherein the SUPRANET intervention arrived as intended, with higher levels of adherence to the guideline recommendations (safety plans, waiting lists, involvement of families/caretakers, continuity of care (measured as staff turnover)), will report better results on all three study outcomes over time compared with institutions that had performed less well.

## 2 Materials and methods

### 2.1 Design

**Figure 1:**
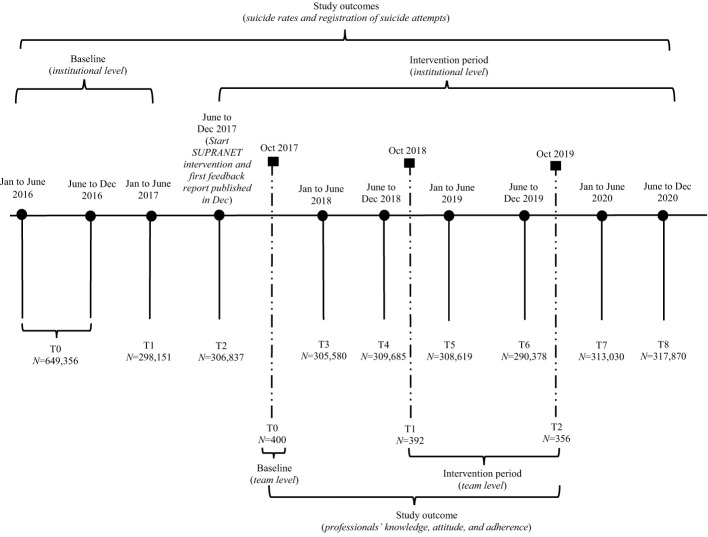
Timeline illustrating the nine measurements performed at the institutional level (suicides and registration of suicide attempts) and the three measurements conducted at the team level (professionals' knowledge, attitudes, and adherence). This figure depicts the total study period (2016 to 2020).

As shown in **Figure 1**, data was collected between January 2016 and December 2020 using an uncontrolled longitudinal prospective design (19). To investigate the effectiveness of the SUPRANET intervention over time on all study outcomes, data was analyzed in three steps. Step 1: Evaluation of the distribution and usage of the SUPRANET intervention (did the intervention arrive as intended?). The arrival of the SUPRANET intervention was measured as follows: 1] how many participants from an institution have read a feedback report, 2] to what extent did institutions distribute the feedback reports organization-wide, and 3] did institutions develop any best practices based on these feedback reports. Step 2: Assessment of the institutions’ compliance with the guideline recommendations (were the SUPRANET quality indicators adequately monitored?). This study included four SUPRANET indicators: 1) having a safety plan, 2) short waiting list duration, 3) family involvement during treatment (measured as registration of contact persons), and 4) continuity of care (measured as staff turnover). These indicators were systematically identified and prioritized in a Delphi study conducted among experts, professionals, and client councils (22). Continuity of care was operationalized as staff turnover, as other aspects of continuity of care were not feasible to monitor. Furthermore, Kapur et al. (4) demonstrated that staff turnover is a very important indicator associated with suicide: Institutions having low rates of staff turnover (especially non-medical staff) showed significantly fewer suicides over time than institutions having high rates of staff turnover. Step 3: The effect of the SUPRANET intervention on all three study outcomes over time (reduced suicide rates, increased registration of suicide attempts, and improved professionals’ knowledge, attitudes, and adherence to the guideline). As previously described, we expect that institutions complying with most of the SUPRANET indicators and performing the intervention as intended will report better results on all three study outcomes than institutions doing less.

This study used a 2-level structure ('institutional' (level 1) and 'team' (level 2)) to collect data on all three study outcomes. For the first two study outcomes (suicide rates and registration of suicide attempts), measurements were performed biannually at the institutional level between Jan 2016 and Dec 2020 (**Figure 1**). Every institution extracted the biannual data from their registration systems based on the Minimal Dataset (MDS; **Supplementary Table S1**). In 2017, there was a change in the MDS regarding the registration of suicides. Starting in 2017, the data collection period was extended by 14 days, asking institutions to not only deliver the number of patients who died by suicide while under their care but also to register how many of their patients had died by suicide within 14 days of discharge or disenrollment.

This biannual data was provided to SUPRANET on an aggregated level (**Supplementary Table S1**). The collected data variables and definitions included: all patients in treatment, suicides, and suicide attempts per gender, age group, psychiatric diagnosis, type of care, and the four SUPRANET quality indicators (safety plans, waiting list duration, involvement of families or caretakers, and staff turnover). In case a patient dies by suicide during treatment in Dutch mental healthcare, this event is always registered and evaluated comprehensively. A predefined format is used to write a report, saved and supervised by a medical director.

Due to the aggregation, no personal patient information could be decrypted by data analysts of SUPRANET (19). For the third study outcome (adherence to the guideline) data was collected annually at the team level (**Figure 1**) using an online questionnaire between Oct 2017 and Oct 2019. A timeline of the SUPRANET study (2016 to 2020) can be found in **Figure 1**.

### 2.2 Ethical approval

This study has been approved by the Central Committee on Research Involving Human Subjects in the Netherlands (CCMO) and does not fall under the scope of the Medical Research Involving Human Subjects Act (WMO). The CCMO states that: ‘In general, research with human subjects only falls under the Medical Research (Human Subjects) Act (WMO) if there is an infringement of the physical and/or psychological integrity of the subject’ (https://english.ccmo.nl/investigators/legalframework-for-medical-scientific-research/your-research-is-it-subject-to-the-wmo-or-not).

### 2.3 Procedure

#### 2.3.1 Recruitment of institutions

In 2016, 13 (out of 25) large mental health institutions in the Netherlands agreed to participate in SUPRANET, providing care to more than 300,000 patients. These institutions were recruited during invitational conferences to inform candidates about the nature of the SUPRANET program. Institutions were considered eligible if they provided specialist care involving acute inpatient clinics, residential care, outpatient clinics, crisis resolution/home treatment care, 24/7 outreaching services, open and closed psychiatric wards to legally apply (forced) care/treatment, and partial hospitalization for adults and elderly (18 years and older).

#### 2.3.2 SUPRANET intervention

**Figure 2:**
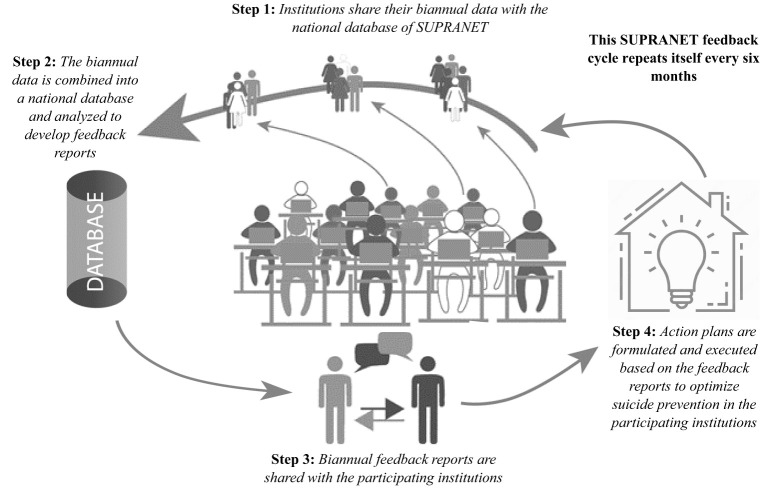
Illustration of the SUPRANET feedback cycle.

The SUPRANET intervention is a national quality improvement initiative based on data collection and data feedback. This initiative aims to improve the implementation of the suicide prevention guideline in Dutch specialist mental healthcare. The SUPRANET intervention is described in more detail in the study protocol (19). Components of the SUPRANET intervention are: 1) developing biannual feedback reports with benchmark information on suicide rates, number of suicide attempts, and the quality indicators [safety planning, waiting time, family involvement (measured as registration of contact persons), and continuity of care (measured as staff turnover)] (19). This benchmark information was given to each institution with the intention that these institutions would formulate and execute action plans. This cycle repeated itself every six months (**Figure 2**). Next, 2) the execution of the second component of the SUPRANET intervention deviated from the study protocol (19). Eventually, we developed intensive bottom-up improvement programs, lasting six to eight months, which were used to optimally support, engage, and involve teams in terms of quality improvement. Programs were focused on quality indicators, such as continuous involvement of families/caretakers and safety plans. The SUPRANET network recommended institutions to participate in these improvement programs. Participating teams received face-to-face educational and learning meetings once every few weeks (every session lasted a few hours). These improvement programs gave teams impulses on how to improve their suicide prevention care (e.g., better involvement of families/caretakers). Best practices were formulated and implemented at the team level. Finally, 3) the third component of the intervention was to organize exchange meetings, educational sessions, webinars, and outreach visits to help the institutions interpret their feedback reports. Helping institutions understand the data in the feedback reports gave them further impulses to formulate action plans for quality improvement. The first and last components of the SUPRANET intervention were primarily delivered at the institutional level. The second component was executed at the team level.

##### 2.3.2.1 Step 1: distribution and usage of the SUPRANET intervention (moderator)

A protocol deviation occurred: a self-report questionnaire was used instead of interviews (**Supplementary Table S2**). A questionnaire was easier to execute and allowed more participants to be included (19). In April 2019, a total of 29 directors, medical directors, board members, chairpersons of suicide prevention boards, and chairpersons of patients’ and families’ advisory boards from the institutions filled out the questionnaire. Participant characteristics are described in **Supplementary Table S2**. This questionnaire consists of three subscales and assesses the distribution and usage of the SUPRANET intervention through 21 items. If participants could not answer an item, a separate option (‘do not know’) was allowed. This questionnaire was used to rate every institution as positive [+], neutral {+/-], or negative [-]. Institutions receiving a positive or neutral rating were labeled as above average for step 1, and those receiving a negative rating were categorized as below average. This procedure was developed *a priori* to the data analysis and is explained in short hereafter.

Every institution began with a positive rating. Subscale 1 measured how many participants from an institution have read a feedback report (i.e., benchmark information on (attempted) suicides and the quality indicators). For subscale 1, institutions could receive one out three scores, that is: no downgrade (100% of the participants have read a report), a downgrade by one (most participants (>50% to <100%) have read a report), or a full downgrade (50% or less of the participants have read a report). Subscale 2 (10 items; five-point Likert scale) measured if mental health institutions adequately distributed the feedback reports. Subscale 3 used nine items (five-point Likert scale) to assess if institutions formulated best practices based on the feedback reports (**Supplementary Table S2**). Item scores for subscale 2 were subsequently summed to generate a total score for each institution separately. The same was done for subscale 3. If a subscale was completed by more than one participant from the same institution, scores were averaged together to one total score per institution. Consequently, every institution received one total score for subscale 2 and another for subscale 3 (**Table 1**). Quartiles were used to assess the distribution of total scores for subscales 2 and 3 [first quartile served as a cut-off (subscale 2 = 26.5, range (12, 47); subscale 3 = 32, range (19, 41))]. For subscale 2, institutions were not downgraded if their total score fell above the cut-off point of 26.5. If the total score fell below the cut-off, it served as a sign that institutions did not properly execute the SUPRANET intervention. Proper usage and dissemination of the reports were considered a prerequisite for subscale 3. If institutions did not meet subscale 2, they were fully downgraded and not assessed on subscale 3. Institutions that did meet subscale 2 were assessed on subscale 3. A depiction of the rating process is shown in **Supplementary Table S3A**.

**Table 1:** Percentage scores for subscale 1 (participants (N=29)) and total scores for subscales 2 and 3 (participants (N=29) and mental health institutions (N=13)) derived from the 21-item questionnaire (step 1).

*Step 1*: Distribution and usage of the SUPRANET intervention		Mental health institutions^1^														
		1	2	3	4	5	6	7	8	9	10	11	12	13		
Subscales	Groups															
		Percentages (%)^4^													Range	
I. Participants reading feedback reports	Participants (directors, medical directors, board members, and chairpersons)	100	100	≤50	100	≤50	100	>50 to <100	>50 to <100	100	100	*ND*	≤50	100	0 - 100	
		Total scores													Range	Cut-off
II. Distribution of feedback reports	Participants (directors, medical directors, board members, and chairpersons)	*26	35	***MD	44	**MD	44	34	**MD	35	*12	*ND*	35	34		
		*24		***MD	*49	***MD	*34	*20	37	44			***MD	*29		
		**MD		***MD			*26	***MD	***MD	44				33		
	Mental health institutions^2,x^	**25**	**35**	*MD*	**47**	*MD*	**35**	**27**	**37**	**41**	**12**	*ND*	**35**	**32**	**10 – 50**	**> 26.5**
		Total scores													Range	Cut-off
III. Formulating best practices (from feedback reports)	Participants (directors, medical directors, board members, and chairpersons)	*32	41	***MD	31	35	32	35	*28	40	19	*ND*	36	34		
		*27		***MD	**MD	***MD	*36	35	*36	39			***MD	32		
		*39		***MD			**MD	***MD	***MD	37				33		
	Mental health institutions^3,x^	**33**	**41**	*MD*	**31**	**35**	**34**	**35**	****32	**39**	**19**	***ND***	**36**	**33**	**9 – 45**	**> 32**

ND, not delivered (participants).

MD, missing data.

The bold values for subscales 2 and 3 are the mental health institutions' total scores (calculated by: adding the total scores of participants and dividing them by the number of total scores).

^1^imputation was performed with 70% of non-missing cases.

^2^cut-off value > 26.5.

^3^cut-off value > 32.

^4^percentage of participants that have read a feedback report.

*scores imputed by using Person Mean Substitution Method (23).

**missing data (participant answered majority of items as ‘do not know’ (>30% of missing cases)).

***missing data (if a participant did not read a feedback report, the questionnaire was automatically closed). Reading a feedback report was a prerequisite for filling out subscales 2 and 3.

****the institution had a borderland score on subscale 3.

##### 2.3.2.2 Step 2: compliance with the SUPRANET quality indicators (moderator)

For step 2, percentages were used to assess every institution’s compliance with the predefined indicators (**Table 2**). We also measured the total network's performance on each indicator (during the intervention period) by calculating four threshold values (safety plans 16.6%, waiting lists 43.4%, involvement of family 20.1%, staff turnover 9.9%; **Table 2**). Institutions received a separate rating for every indicator [positive [+], neutral [+/-], negative [-]; **Supplementary Table S3B**]. Institutions were assessed on three criteria.

**Table 2:** Percentages illustrating the institutions’ compliance with the SUPRANET quality indicators over time (step 2).

*Step 2*: Compliance with the SUPRANET quality indicators	Mental health institutions													Total network (*SUPRANET*)
	1	2	3	4	5	6	7	8	9	10	11	12	13	
Safety plans	Percentages (%)													Threshold (%)^x^
Improved? *(baseline vs intervention)*	**MD**	**No**	**Yes**	**Yes**	**Yes**	**Yes**	**No**	**No**	**Yes**	**Yes**	*NM*	*NM*	*NM*	
Baseline (*T0* and *T1*)	MD^a^	21.0	5.5	11.2	3.8	7.4	28.1	32.5^c^	16.4	21.3	*NM*	*NM*	*NM*	
Intervention (*T2* to *T8*)	25.7^b^	20.7	11.2	12.3	14.6	9.1	19.8	27.0	17.9	27.9	*NM*	*NM*	*NM*	**16.6**
registration of contact persons	Percentages (%)													Threshold (%)^x^
Improved? *(baseline vs intervention)*	**MD**	**Yes**	**Yes**	**No**	**Yes**	**Yes**	**Yes**	**Yes**	**Yes**	**Yes**	**Yes**	**Yes**	**MD**	
Baseline (*T1*)*	MD^d^	41.9	96.4	89.9	21.0	32.0	50.7	50.3	57.5	44.9	46.8	29.0	MD***	
Intervention (*T2* to *T8*)	20.3^e^	65.1	96.7	73.6	25.5	33.8	62.6	67.2	59.7	54.9^e^	59.2	33.5**	55.0	**43.4**
Waiting list duration	Percentages (%)													Threshold (%)^x^
Improved? *(baseline vs intervention)*	**Yes**	**No**	**Yes**	**Yes**	**Yes**	*NM*	**No**	**Yes**	**Yes**	**No**	**Yes**	**Yes**	**Yes**	
Baseline (*T0* and *T1*)	14.0	13.6	17.9	29.5	6.6	*NM*	16.1	21.6^f^	10.0	74.2	24.9	9.7	14.9***	
Intervention (*T2* to *T8*)	18.2	11.8	18.0	30.8	15.6	*NM*	14.4	23.5	10.8	64.1	25.0	10.8**	33.2	**20.1**
Staff turnover	Percentages (%)													Threshold (%)^x^
Improved? *(baseline vs intervention)*	**No**	**Yes**	**No**	**No**	**Yes**	**No**	**No**	**No**	**No**	**Yes**	**No**	**No**	**MD**	
Baseline (*T1*)*	7.8	9.5	9.2	5.9	16.7	5.3	3.9	6	5.7	15.3	1.8	9.2	MD***	
Intervention (*T2* to *T8*)	7.9	5.0	10.5	20.2	12.7	6.6	6.1	13.5	8.7	14.8	6.1^g^	9.7**	7.6	**9.9**

MD, missing data.

NM, indicator not monitored throughout the study period.

^x^SUPRANET's threshold values for safety plans, involvement of families/caretakers, waiting lists, and staff turnover (int period). Calculation thresholds (int period): the percentages of the institutions were summed and weighed into a single value for each indicator.

*data collection of the indicator started at T1.

**indicator was not monitored at T6.

***the dataset provided by the institution (T1) contained technical errors. The institution could not properly extract the requested data due to unforeseen issues with its new data warehouse system. To minimize possible bias: erroneous data (registration of contact persons/waiting lists/staff turnover) was not included in the rating procedure for step 2 (criteria 1, 2, and 3) and was also removed from further analyses. Data provided by the institution on safety plans (T1) was deemed valid and therefore included in the rating procedure.

a institution did not monitor indicator (safety plans) at T0 and T1.

b institution did not monitor indicator (safety plans) at T2.

c institution did not monitor indicator (safety plans) at T0.

d institution did not monitor indicator (registration contact persons) at T1.

e institution did not monitor indicator (registration contact persons) at T2.

f institution did not monitor indicator (waiting lists) at T0.

g institution incorrectly monitored indicator (staff turnover) at T3.

Similar to step 1, every institution began with a positive rating. The first criterion measured if institutions monitored an indicator on every biannual timepoint, receiving no downgrade (monitored on every biannual timepoint), one downgrade (not monitored on every timepoint), or a full downgrade (not monitored at all). For criterion 2, institutions had to monitor the indicator better than the total network during the intervention period. SUPRANET’s threshold values were used to assess if institutions met criterion 2 (**Supplementary Table S4**; **Table 2**). Institutions received no downgrade (indicator was monitored above the threshold value) or one downgrade (indicator was monitored below the threshold value). The fourth indicator 'staff turnover' was rated inversely (i.e., institutions with low rates of staff turnover were generally perceived as more stable (4)). If institutions did not meet criterion 2, they were assessed on the third criterion (i.e., has the institution made any efforts to improve its compliance with the indicator over time? (**Table 2**; **Supplementary Table S4**)). If criterion 3 was also not met, institutions received a full downgrade. A depiction of the rating process is shown in **Supplementary Table S3B**. As already mentioned, mental health institutions received a separate rating for each of the four indicators.

These four ratings were used to label every mental health institution as above average or below average for step 2. The procedure described in **Supplementary Table S3B** was developed prior to the data analysis.

##### 2.3.2.3 Step 1 and step 2 combined

After combining steps 1 and 2, five institutions received a final score of above average because they executed the intervention more as intended (step 1) and better monitored the indicators (step 2). The remaining eight institutions were in the below-average group (**Supplementary Table S4**).

##### 2.3.2.4 Statistical analyses and measurements

ITSA is suitable for data collected at an aggregated level and accounts for autocorrelated data (24). A three-step interrupted time series analysis (ITSA) was conducted to investigate the effect of SUPRANET on the first two study outcomes: 1) standardized mortality ratio for suicide (SMR) and 2) registration of suicide attempts. Firstly, a single-group ITSA was performed to examine the effect of the intervention on the total network (*N*=13). Secondly, a multiple-group ITSA was conducted to investigate if institutions labeled as above average reported better results on the two study outcomes over time than institutions assigned as below average. The third outcome (professionals’ knowledge and adherence) was analyzed as a continuous variable by performing a longitudinal multilevel regression analysis. A sum score was calculated for each scale. We adjusted for working experience (in years), profession, completing a suicide prevention training (yes/no), and team (1=crisis team, 0=ambulatory care teams) as confounders. The classification of the institutions (above average/below average) was included as a moderator (independent variable) in the longitudinal multilevel regression analyses and in the ITSA analyses. All statistical analyses were performed using SPSS version 24.0 or Stata 15.0. All reported *p*-values are two-tailed, with a 95% confidence interval (95%CI), and *p*<0.05 was the threshold for statistical significance. Missing data was imputed using the person mean substitution method, using a cut-off value of at least 70% non-missing values (23).

#### 2.3.3 SUPRANET intervention period versus baseline

##### 2.3.3.1 Suicide rates (institutional level)

Firstly, the overall trend in suicides was investigated by comparing the baseline (T0-T1: institutional level (**Figure 1**)) and intervention period across all 13 institutions. For this study outcome, we used T2 as the starting point of the intervention period (**Figure 1**). Secondly, we analyzed whether we could identify a reduction in suicide rates over time (baseline vs intervention) by comparing above-average institutions versus below-average institutions. To analyze trends in suicides, we adjusted suicide rates for confounding factors in the client population (e.g., demographic and psychiatric severity factors) by using indirect standardization, also known as standardized mortality rates (SMRs) (25). Adjusting for risk factors like gender, age, setting, and psychiatric diagnosis by calculating SMRs biannually made it possible to compare the data between institutions.

##### 2.3.3.2 Registration of suicide attempts (institutional level)

The secondary study outcome is the extent to which suicide attempts were being registered. Again, we used T2 as the starting point of the intervention period (**Figure 1**) to investigate if there was an increase in the registration of suicide attempts across all 13 institutions over time, as well as between the two groups of institutions (above average vs below average). To analyze this study outcome, we calculated the number of registered suicide attempts per 100,000 patients.

##### 2.3.3.3 Professionals’ knowledge and adherence to the guideline (team level)

This study outcome was measured annually using a self-report questionnaire (**Figure 1**), including a 7-item subscale measuring professionals’ knowledge (5-point Likert scale) and a 3-item subscale for professionals’ attitudes (5-point Likert scale). Both subscales were based on a questionnaire used in the Professionals In Training to Stop Suicide (PITSTOP) study (13). Professionals' adherence is an 8-item subscale (5-point Likert scale; 1=(almost) never to 5=always), based on items from the Zero Suicide Monitor (ZSM) (26). The self-report questionnaire was distributed among 12 institutions. One institution was not included in the analysis because this organization had already adopted its own version of the ZSM among its employees. Professionals were asked to fill out demographic information (profession, working experience, and for which institution they worked). Professionals answered how many patients they had treated with suicidal thoughts and behaviors during the past month (0-100 patients) and if they had participated in a suicide prevention training (26). We analyzed if 1) there was an increase for the total network (*N*=13) in professionals’ knowledge, attitudes, and adherence over time (using T1 as the starting point of the intervention period (see **Figure 1**)), and 2) if there were differences in professionals’ knowledge, attitudes, and adherence to the guideline between the above-average-group and between average and group.

## 3 Results

### 3.1 Characteristics of institutions

At baseline (T0-T1: institutional level (**Figure 1**)), the 13 organizations provided specialist mental healthcare (S-GGZ) to the majority of their patients (84.8%; **Supplementary Table S5**). Every participating institution provided care to adults and older people (>18 years), whereas the majority of institutions (*n*=12) also provided pediatric mental health services (for children and adolescents up to the age of 18 years). Across all institutions, most patients were being treated for mood disorders (19.7%), followed by anxiety disorders (13.0%). Every year, the network (*N*=13) provided care to more than 300,000 patients across the Netherlands. The number of patients cared for by every institution on a half-yearly basis typically ranged between 8,400 and 33,400. However, one institution stood out, providing care to at least 100,000 patients every half year, making it the largest institution in the dataset. From 2016 to 2020, a total of 1,686 patients died by suicide within the participating institutions. Characteristics of all patients in care at baseline are illustrated in **Supplementary Table S5**.

### 3.2 Professional characteristics

The sample at baseline (T0: team level (**Figure 1**)) consisted of 400 mental health professionals working in 22 teams (10 ambulatory care teams and 12 crisis teams) among the 12 institutions (**Supplementary Table S6**). Most professionals worked in crisis teams (*n*=246) compared with ambulatory care teams (*n*=154). Overall (*N*=400), most professionals had >10 years of working experience (67.8%). Most professionals were working as specialized social psychiatric nurses (26.3%), psychiatrists (18.0%), or nurses (17.8%). Professionals working in crisis teams treated patients with suicidal thoughts and behaviors more often (M=15.35; SD=13.1) compared with professionals working in ambulatory care teams (M=9.03; SD=9.37). In crisis teams, more participants were trained in suicide prevention (79.7%) compared with participants in ambulatory care teams (66.9%).

### 3.3 SUPRANET intervention period versus baseline

#### 3.3.1 Suicide rates (institutional level)

For the total set of mental health institutions, we found a significant biannual decrease in suicides (SMR) prior to the start of the SUPRANET intervention [b= -0.076 per half year, *p*<0.001, 95%CI=(-0.099, -0.053)]. However, directly after the start of the intervention (T2), an increase in suicides of 0.156 occurred [*p*<0.001, 95%CI=(0.102, 0.210)]. Throughout the rest of the study period, the biannual trend in suicides decreased again (**Supplementary Figure S1**). However, its slope was statistically significantly less steep compared with the period before the SUPRANET intervention [b=0.057 per half year, *p*=0.002, 95%CI=(0.029, 0.085)].

**Figure 3:**
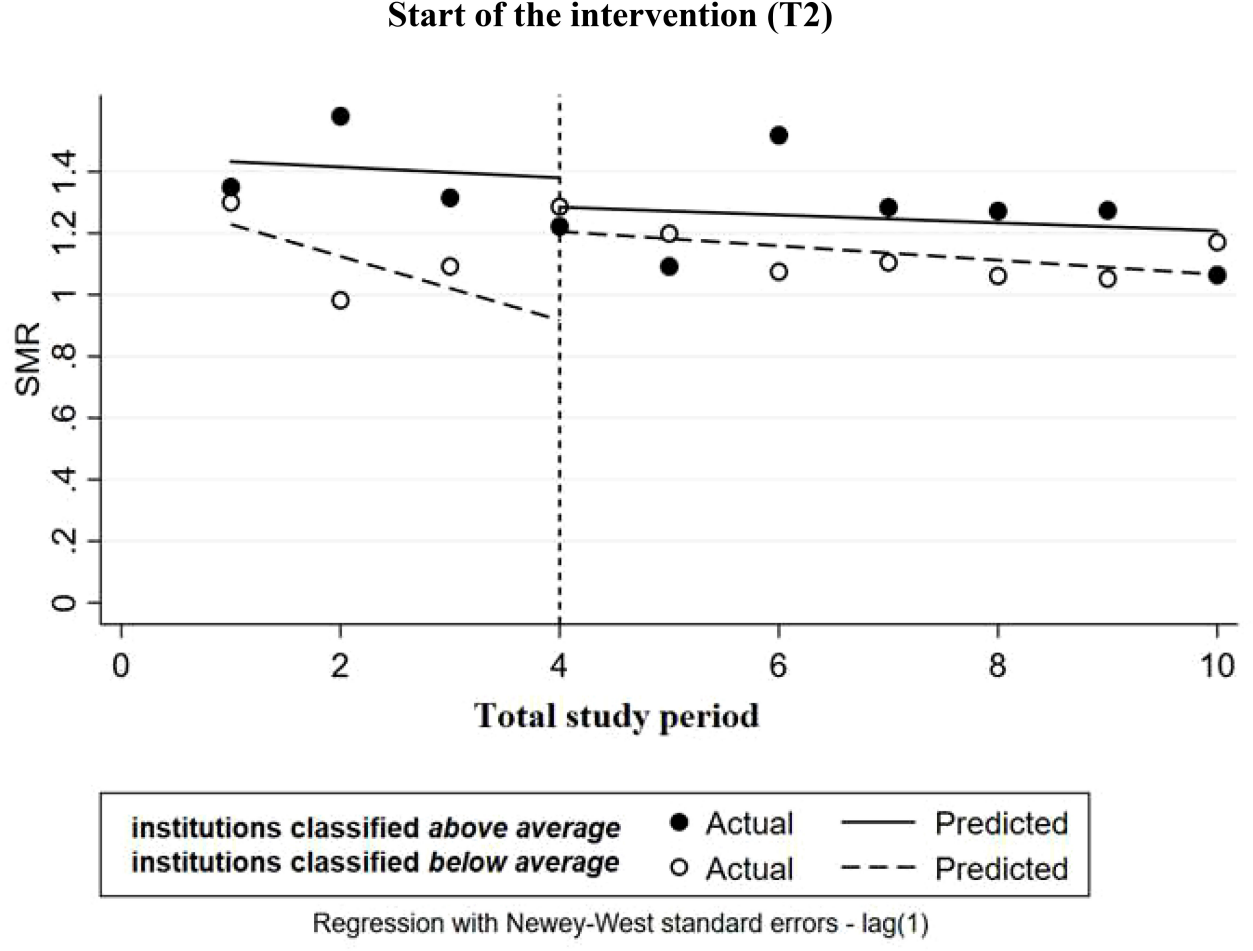
Multiple-group ITSA on suicides (measured as: standardized mortality ratio (SMR)) with Newey-West standard errors and one lag (institutions labeled as *above average* or *below average*). The institutions are comparable at the baseline level and trend.

After institutions were labeled based on their efforts and investments made (above average vs below average), only institutions labeled as below average showed an increase in suicides directly after the start of the intervention. Although this increase was not statistically significant, it does suggest a potential trend [b=0.287, *p*=0.069, 95%CI=(-0.029, 0.606); **Figure 3**]. Furthermore, no statistically significant difference in suicides between the two groups was found directly after the start of the intervention [b= -0.383, *p*=0.112, 95%CI=(-0.869, 0.103)], nor were any significant differences detected throughout the rest of the study period [(**Figure 3**); b= -0.075 per half year, *p*=0.503, 95%CI=(-0.314, 0.163)].

#### 3.3.2 Registration of suicide attempts (institutional level)

For the total set of mental health institutions, we found no statistically significant effect on the registration of suicide attempts directly after the start of the SUPRANET intervention [5.4 per 100,000 patients, *p*=0.548, 95%CI= (-15.3 per 100,000, 26.0 per 100,000); **Supplementary Figure S2**]. As the study progressed, we did detect a statistically significant increase in the biannual trend of 8.1 registered suicide attempts per 100,000 patients compared with the period before the SUPRANET intervention [*p*=0.044, 95%CI=(0.3 per 100,000, 15.9 per 100,000)].

**Figure 4:**
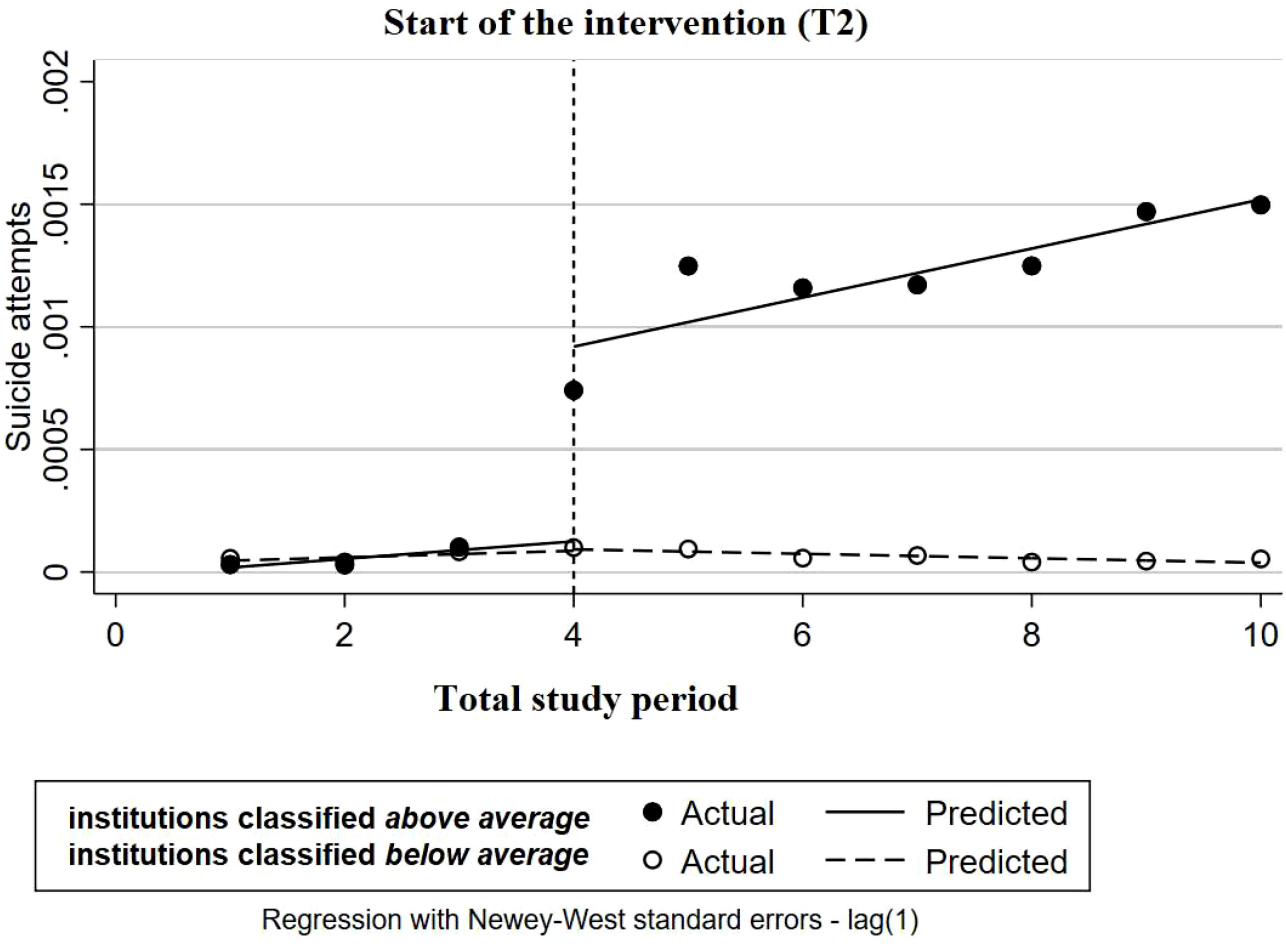
Multiple-group ITSA for proportion of patients with reported suicide attempts with Newey-West standard errors and one lag (institutions labeled as *above average* or *below average*). The institutions are comparable at the baseline level and trend.

After labeling institutions as below average or above average, a differential pattern of change in the baseline was detected in terms of intercept [-27.4 per 100,000, *p*<0.001, 95%CI=(-31.7 per 100,000, -23.1 per 100,000)] and slope [4.5 per 100,000, *p*=0.019, 95%CI=(0.85 per 100,000, 8.1 per 100,000)]. To make groups comparable three institutions were removed (**Figure 4**) (21). We found that institutions labaled as above average registered significantly more suicide attempts directly after the start of the intervention [78.8 per 100,000 patients, *p*<0.001, 95%CI=(51.3 per 100,000, 106.4 per 100,000)], and throughout the rest of the study period, they continued to perform significantly better on this outcome, reporting an increase in the biannual trend of 8.7 registered attempts per 100,000 patients [**Figure 4**; *p*=0.004, 95%CI=(3.3 per 100,000, 14.1 per 100,000)].

#### 3.3.3 Professionals’ knowledge, attitudes, and adherence to the guideline (team level)

For the total network, a significant increase in professionals' knowledge [b=0.54, *p*<0.001, 95%CI=(0.23, 0.84)] and guideline adherence was found over time compared to the baseline [b=1.82, *p*<0.001, 95%CI=(1.13, 2.51)]. For the attitude scale, no significant changes were found.

After categorizing institutions as above average or below average, we found no statistically significant difference in professionals' knowledge between the two groups over time. (**Table 3**; **Supplementary Figure S3**). However, we did find a stronger improvement in professionals’ attitudes for institutions labeled as below average at T1 (**Table 3**; **Supplementary Figure S4**) [b= -0.38, *p*=0.004, 95%CI=(-0.65, -0.12)], but this effect did not remain significant at the end of the study period [b=0.007, *p*=0.956, 95%-CI=(-0.26, 0.27)]. Lastly, we found that institutions classified as above average significantly better adhered to the suicide prevention guideline over time (**Supplementary Figure S5**) [b=1.39, *p*=0.032, 95%CI=(0.12, 2.65)].

**Table 3:** Differences in professionals’ knowledge, attitudes, and adherence to the guideline between institutions labeled as *above average* (complied with the indicators and performed the SUPRANET intervention more as intended) and those labeled as *below average* over time.

	Model 1: Unadjusted model				Model 2: Adjusted model			
	Diff.^1^	95%CI		*p*-value	Diff.^1^	95%CI		*p*-value
		Lower bound	Upper bound			Lower bound	Upper bound	
Study outcomes^2,3^								
Professionals’ knowledge								
*T0*	-0.06	-0.69	0.57	0.845	-0.02	-0.60	0.56	0.947
*T1*	0.39	-0.25	1.02	0.229	0.52	-0.06	1.10	0.080
*T2*	-0.14	-0.79	0.50	0.664	-0.04	-0.64	0.55	0.882
Professionals’ attitudes								
*T0*	0.03	-0.24	0.30	0.825	0.09	-0.17	0.35	0.489
*T1*	-0.51	-0.78	-0.24	**<0.001**	-0.38	-0.65	-0.12	**0.004**
*T2*	-0.12	-0.40	0.15	0.368	0.007	-0.26	0.27	0.956
Professionals’ adherence								
*T0*	0.48	-0.74	1.70	0.441	0.27	-0.96	1.51	0.665
*T1*	-0.11	-1.37	1.16	0.867	-0.33	-1.62	0.96	0.619
*T2*	1.49	0.26	2.74	**0.018**	1.39	0.12	2.65	**0.032**

^1^A negative value implicates that the institutions in the above-average group scored lower on the study outcome compared with those in the non-adherent group.

^2^Institutions complying with the indicators and performing the SUPRANET intervention more as intended (above average) versus those doing less (below average).

^3^The below-average category was used as the reference group. For time: T0 (baseline) was used as the reference group.

Values in bold indicate statistically significant results.

## 4 Discussion

This is the first longitudinal study reporting on the effects of an internationally unique initiative called SUPRANET. This study aimed to evaluate the impact of the impact of SUPRANET on three study outcomes, whereby data was collected on a 2-level structure (institutional- and team level). Firstly, after categorizing institutions based on their efforts and investments made (above average vs below average), analyses showed no superior intervention effect on suicides for one group of institutions over the other. Secondly, we found that institutions complying with the SUPRANET indicators and performing the intervention as intended, significantly better registered suicide attempts over time than institutions that had performed less well. Thirdly, the above-average group significantly better adhered to the guideline over time than the below-average group.

In this study, we found some unexpected results. For the total network, a significant decrease in suicides was found during the baseline period, however, within a few months after the start of the SUPRANET intervention a significant increase in suicides was detected. A possible explanation for finding these contradictory results may be because of registration issues and underreporting of suicides prior to the study. It is plausible that after the start of SUPRANET, institutions became more aware of the urgency to register their data, possibly leading to an improved registration effect. This assumption is further strengthened due to the fact that this effect was only found for the group of institutions that had performed less well in terms of suicide prevention (**Figure 3**), suggesting that these institutions already had less-than-optimal data registration prior to the study period, and improved their data registration after joining the network. Secondly, compared to 2016, the operationalization of suicides as a study outcome was slightly changed in the Minimal Dataset (MDS) from 2017 onwards, asking institutions to not only deliver the number of patients who died by suicide while currently under their care but also to register how many of their patients had died by suicide within 14 days of discharge or disenrollment, and who were not yet in care at another institution. This change in the MDS of extending the data collection period by 14 days might also be an explanation for finding a biannual increase in the number of suicides.

Preventing suicides is complex and multifactorial. The key preventive strategies influencing suicide risk likely go wider than those implemented and evaluated in our study, considering that we only included four quality indicators, and used one self-report questionnaire to evaluate the institutions’ uptake of the SUPRANET intervention. It is plausible that other unmeasured confounding factors also might influence the SMRs, such as shrinkage of psychiatric bed base over time (by government force), regional differences, and the fluctuating nature of (bi)annual suicide rates. Furthermore, according to the CANS data (27) and the Dutch Health Inspectorate (1), the number of people who have died by suicide in the Netherlands has not decreased within the study period. Knowing this, it would not have been very likely for our study to find a convincing drop in the number of suicides, as this would have been contradictory to the overall trend found by the CANS (27) and the Dutch Health Inspectorate (1).

## 5 Strengths and limitations

The SUPRANET study faced several limitations regarding the implementation of the SUPRANET intervention. We want to address these limitations based on the theoretical model of Grol & Wensing (10). This model suggests that successful intervention implementation should occur at three levels (organizational, team, and patient level).

Firstly, a problem with the setup of this study was that, despite the effort, the feedback reports were not always adequately distributed, meaning that the visibility of these reports was not always successfully established. Although we found that mental health professionals got more familiar with SUPRANET over time (increase from 16% to 35%), most clinicians (about 65%) remained uninformed about the network’s purpose and its activities by the end of the study period. Possible explanations for detecting large differences between institutions in distributing these feedback reports might be that a) the feedback reports were mainly disseminated top-down instead of bottom-up, only reaching board members, directors, and medical directors. As a result, most feedback reports did not reach the work floor (team managers, clinicians). Furthermore, b) not all board members, directors, and medical directors distributed these reports within their organization, as they argued that these reports could have been more meaningful if its benchmark information was tailored to individual teams and not only targeted at the organizational level. In other words, they would have preferred to receive feedback adapted to situations they were responsible for. Because these reports did not align with their needs or wishes, participants were less likely to engage with them. In light of Grol & Wensing’s model (10), we suggest that future studies focus on collecting the data at the team level or patient level, instead of organization-wide. Finally, c) some institutions had less capacity or significant other responsibilities, and therefore, they were less able to distribute, interact, and respond to the feedback. Consequently, some institutions only passively disseminated the feedback reports, but ‘passive’ strategies often lead to little or no effect, and to establish actual change, institutions should start to use more ‘active’ implementation strategies instead. Thus, having several highly motivated clinicians or employees to keep suicide prevention a priority within the institution is essential. Despite busy schedules, lack of time, and other priorities, every institution should feel a sense of urgency, fully support the SUPRANET approach, and share the same vision of investing in suicide-safer care. Secondly, the possible effects caused by the feedback reports might have been ‘too distal’ from the collected data, which could have hindered achieving attitudinal change among mental health professionals. Thirdly, the quality of the collected data depended greatly on the existing registration systems used by each institution. Because the content of most electronic health registration systems (EHRs) was not primarily designed to fully support treatment trajectories with a primary focus on suicidal risk, it was not always feasible -or possible for institutions to extract the desired data. Institutions therefore need to improve their existing data registration systems to overcome these problems, however, not all institutions are equally capable of making these changes. These limitations illustrate the complexity of changing such practices in daily routine. Even among institutions categorized as above average, certain problems and difficulties still occurred, such as less-than-optimal monitoring of quality indicators or not performing all components of the SUPRANET intervention as intended. Because not one institution within the network received an optimal score (for step 1 and step 2), both groups (above average and below average) tend to differ relatively little from each other, reducing the likelihood of finding large significant effects on the study outcomes. The SUPRANET intervention lasted for four years. Knowing this, we should ask ourselves if this timeframe was sufficient enough to find convincing results on the study outcomes (especially reduced suicide rates), or if institutions should commit to SUPRANET for a longer period.

We also faced several shortcomings concerning the study itself. Firstly, although variables were clearly defined and operationalized in the MDS, not all institutions delivered biannual data according to this document. For example, some institutions did not deliver all their ongoing safety plans, leading to a biased representation. To avoid such errors, data management built in extra biannual checks to inspect the quality of the data delivered from institutions after each data collection period. Although this led to some improvements, the quality of the data was often still not optimal. Secondly, using a multi-strategy approach is seen as a promising approach to prevent suicides effectively (4, 7, 14). However, this position only holds when as many evidence-based strategies as possible are selected and implemented simultaneously, as literature shows that this combination leads to the biggest reduction in suicides (4, 14). Although the evidence is building for several strategies, such as safety plans (3), other indicators, including family involvement or short waiting lists, remain understudied. Next to that, the selection of only four indicators in this study might have been insufficient to achieve an actual drop in suicide deaths, given that While et al. (14) recommend to include at least 7 to 9 indicators to have the biggest impact on suicides. Several recommendations are not (yet) monitored by SUPRANET, but have been identified by research in the UK as useful strategies in relation to suicide rates, such as early follow-up after discharge, staff training, 24/7 crisis department, and ward safety (4). Even though almost all institutions in the Netherlands have a 24/7 crisis department, or invest inward safety, limitations of EHRs hinder institutions from systematically registering this data. Thirdly, the data was collected at an aggregated level. Particularly for this study, the data appeared sufficient to answer all research questions. However, future studies might try to take the next step into collecting data at the patient level to analyze and identify patterns over time, for example, by using text mining techniques. Fourthly, regarding the methodology for Interrupted Time Series Analysis (ITSA), it should be noted that this methodology was developed for comparing subjects (in our case: institutions) and not for comparing groups of subjects. However, we used the ITSA methodology to make a distinction between institutions classified as above average (complied with the quality indicators and performed the SUPRANET intervention more as intended) and institutions classified as below average. As noted in the literature, there is a significant methodological gap in ITSA involving aggregated data, where analyses involving such data did not account for heterogeneity (28). Fifth and finally, SUPRANET has not (yet) reached national coverage, considering that from the 25 large institutions in the Netherlands (7), 16 are currently participating. However, because institutions only joined SUPRANET based on their own free will, this might have introduced possible sampling bias.

Next to these limitations, there were also strengths. To our knowledge, SUPRANET is (inter)nationally a unique initiative, conducted on a large scale, including at least 300,000 patients from 13 large Dutch institutions nationwide. Throughout the years, SUPRANET collected and analyzed lots of data at the organizational -and patient level, building an extensive database, making it possible to pool longitudinal data on important healthcare aspects such as safety planning, involvement of families, waiting lists, and staff turnover. By using this enriched database, participating institutions were able to benchmark themselves against each other, gaining more insight into the quality of care they provided to patients with suicidal urges. In this particularly controversial area, SUPRANET is the first benchmark study to try to shed more light on the current state of Dutch clinical practice and how to better implement the guideline recommendations in mental healthcare, especially given the fact that many patients with suicidal urges still do not receive the best possible care there is.

## 6 Conclusion and clinical implications

To significantly reduce the number of suicides in Dutch specialist mental healthcare, institutions across the country joined SUPRANET. Although this study faced several shortcomings (e.g., data registration was less-than-optimal, and investments in the actual execution of the SUPRANET activities were not optimal), we found some promising results. For example, we noticed that institutions ‘doing more’ (i.e., SUPRANET intervention was executed more as intended and the indicators were better monitored), performed better on 2 out of 3 study outcomes over time. These findings further strengthen the argument that the SUPRANET approach has led to these actual changes. We recommend implementing and monitoring quality improvement strategies at the team- and patient level, thereby collecting the data ‘as close’ as possible to the intervention. This study was conducted in a relatively short period, mainly at the organizational level, making it difficult to adequately monitor attitudinal change to the guideline among clinicians. Ideally, all institutions should implement evidence-based or practice-based suicide prevention recommendations into clinical practice, expecting to result in suicide-safer care for all patients in mental healthcare.

## Data availability statement

The raw data supporting the conclusions of this article will be made available by the authors, without undue reservation.

## Ethics statement

This study is part of the SUPRANET project and has been approved by the Central Committee on Research Involving Human Subjects in the Netherlands (CCMO). Although written informed consent was not required in accordance with the Medical Research Involving Human Subjects Act (WMO), consent of participants was given by filling out the survey. Data of participants were anonymized prior to statistical analysis.

## Author contributions

GF, RG, AB, and KS contributed to the design of the study. KS conducted the study under the supervision of RG and AB. KS drafted the manuscript. RG, RW, MV, and AB made critical revisions and edited the manuscript. AH and KS prepared and conducted the statistical analyses. All authors contributed to the article and approved the submitted version.
