# Supplementary material for: Reducing suicides in mental healthcare: results from a 4-year follow-up implementation study in the Netherlands (SUPRANET)
Source: Front Psychiatry. 2024 Apr 19;15:1080235. doi: 10.3389/fpsyt.2024.1080235 (PMC11068092; doi:10.3389/fpsyt.2024.1080235)
Supplement: Supplementary file 1 [file DataSheet_1.zip › Supplementary Table 4.docx]

|  | **Step 1: Distribution and usage of feedback reports** | | | | | **Step 2: Compliance with the SUPRANET quality indicators** | | | | | | | | | | | | | | | | | **Step 1 & step 2 (*overall classification*)** |
| --- | --- | --- | --- | --- | --- | --- | --- | --- | --- | --- | --- | --- | --- | --- | --- | --- | --- | --- | --- | --- | --- | --- | --- |
|  |  |  |  | **Result** | | **Safety plan** | | | | **Registration of contact persons (*involving family & caretakers*)^1^** | | | | **Waiting list duration** | | | | **Staff turnover^1^** | | | | **Result** |  |
|  | **Subscale 1** | **Subscale 2** | **Subscale 3** |  |  | **Criterion 1** | **Criterion 2** | **Criterion 3** |  | **Criterion 1** | **Criterion 2** | **Criterion 3** |  | **Criterion 1** | **Criterion 2** | **Criterion 3** |  | **Criterion 1** | **Criterion 2** | **Criterion 3** |  |  |  |
| Institution | How many individuals have read a feedback report (%)? | Feedback reports distributed as intended (*>26.5*)? | Were best practices formulated (*>32*)? | Rating | Classification step 1 | Indicator monitored on every time point (*yes/no*)? | Indicator adequately monitored during int period (*> 16.6%*)?*** | Improved (baseline vs int; *yes/no*)? | Rating | Indicator monitored on every time point (*yes/no*)? | Indicator adequately monitored during int period (> *43.4%*)?*** | Improved (baseline vs int; *yes/no*)? | Rating | Indicator monitored on every time point (*yes/no*)? | Indicator adequately monitored during int period (*> 20.1%*)?*** | Improved (baseline vs int; *yes/no*)? | Rating | Indicator monitored on every time point (*yes/no*)? | Indicator adequately monitored during int period (*< 9.9%*)?*** | Improved (baseline vs int; *yes/no*)? | Rating | Classification step 2 |  |
| 1 | 100 | No | *NA* |  |  |  |  |  | **+/-** |  | No |  | **-** |  | No |  | **+/-** |  | Yes |  | **+** | **below average** | **below average** |
|  |  |  |  |  |  |  |  |  |  |  |  |  |  |  |  |  |  |  |  |  |  |  |  |
|  |  |  |  | **-** | **below average** | No^a^ | Yes | *NA* |  | No^c^ |  | *MD* |  | Yes |  | Yes |  | Yes |  | *NA* |  |  |  |
| 2 | 100 | Yes | Yes | **+** | **above average** | Yes | Yes | *NA* | **+** | Yes | Yes | *NA* | **+** | Yes | No | No | **-** | Yes | Yes | *NA* | **+** | **above average** | **above average** |
| 3 | <50 | *NA* | *NA* |  |  |  |  |  | **+/-** |  | Yes |  | **+** |  | No |  | **+/-** |  | No |  | **-** | **below average** | **below average** |
|  |  |  |  |  |  |  |  |  |  |  |  |  |  |  |  |  |  |  |  |  |  |  |  |
|  |  |  |  | **-** | **below average** | Yes | No | Yes |  | Yes |  | *NA* |  | Yes |  | Yes |  | Yes |  | No |  |  |  |
| 4 | 100 | Yes | No |  |  |  |  |  | **+/-** |  | Yes |  | **+** |  | Yes |  | **+** |  | No |  | **-** | **above average** | **above average** |
|  |  |  |  | **+/-** | **above average** | Yes | No | Yes |  | Yes |  | *NA* |  | Yes |  | *NA* |  | Yes |  | No |  |  |  |
| 5 | <50 | *NA* | *NA* |  |  |  |  |  | **+/-** |  | No |  | **+/-** |  | No |  | **+/-** |  | No |  | **+/-** | **below average** | **below average** |
|  |  |  |  | **-** | **below average** | Yes | No | Yes |  | Yes |  | Yes |  | Yes |  | Yes |  | Yes |  | Yes |  |  |  |
| 6 | 100 | Yes | Yes |  |  |  |  |  | **+/-** |  | No |  | **+/-** |  | *NM* |  | **-** |  | Yes |  | **+** | **below average** | **below average** |
|  |  |  |  |  |  |  |  |  |  |  |  |  |  |  |  |  |  |  |  |  |  |  |  |
|  |  |  |  | **+** | **above average** | Yes | No | Yes |  | Yes |  | Yes |  | *NM* |  | *NM* |  | Yes |  | *NA* |  |  |  |
| 7 | >50 to <100 | Yes | Yes |  |  |  |  |  | **+** |  | Yes |  | **+** |  | No |  | **-** |  | Yes |  | **+** | **above average** | **above average** |
|  |  |  |  |  |  |  |  |  |  |  |  |  |  |  |  |  |  |  |  |  |  |  |  |
|  |  |  |  | **+/-** | **above average** | Yes | Yes | *NA* |  | Yes |  | *NA* |  | Yes |  | No |  | Yes |  | *NA* |  |  |  |
| 8 | >50 to <100 | Yes | ****Yes |  |  |  |  |  | **+/-** |  | Yes |  | **+** |  | Yes |  | **+/-** |  | No |  | **-** | **below average** | **below average** |
|  |  |  |  |  |  |  |  |  |  |  |  |  |  |  |  |  |  |  |  |  |  |  |  |
|  |  |  |  | **+/-** | **above average** | No^b^ | Yes | *NA* |  | Yes |  | *NA* |  | No^e^ |  | *NA* |  | Yes |  | No |  |  |  |
| 9 | 100 | Yes | Yes |  |  |  |  |  | **+** |  | Yes |  | **+** |  | No |  | **+/-** |  | Yes |  | **+** | **above average** | **above average** |
|  |  |  |  |  |  |  |  |  |  |  |  |  |  |  |  |  |  |  |  |  |  |  |  |
|  |  |  |  | **+** | **above average** | Yes | Yes | *NA* |  | Yes |  | *NA* |  | Yes |  | Yes |  | Yes |  | *NA* |  |  |  |
| 10 | 100 | No | *NA* | **-** | **below average** | Yes | Yes | *NA* | **+** | No^d^ | Yes | *NA* | **+/-** | Yes | Yes | *NA* | **+** | Yes | No | Yes | **+/-** | **above average** | **below average** |
| 11 | *ND* | *ND* | *ND* | **-** | **below average** | *NM* | *NM* | *NM* | **-** | Yes | Yes | *NA* | **+** | Yes | Yes | *NA* | **+** | No^f^ | Yes | *NA* | **+/-** | **above average** | **below average** |
| 12 | <50 | *NA* | *NA* |  |  |  |  |  | **-** |  | No |  | **-** |  | No |  | **-** |  | Yes |  | **+/-** | **below average** | **below average** |
|  |  |  |  | **-** | **below average** | *NM* | *NM* | *NM* |  | No* |  | Yes |  | No* |  | Yes |  | No* |  | *NA* |  |  |  |
| 13 | 100 | Yes | Yes | **+** | **above average** | *NM* | *NM* | *NM* | **-** | Yes | Yes | *NA* | **+**** | Yes | Yes | *NA* | **+**** | Yes | Yes |  | **+**** | **above average** | **above average** |
|  |  |  |  |  |  |  |  |  |  |  |  |  |  |  |  |  |  |  |  | *NA* |  |  |  |

**Supplementary Table 4.** Overview classification institutions for step 1 (distribution and usage of the SUPRANET intervention) and step 2 (compliance with the quality indicators).

MD = missing data.

NA = institution not assessed on subscale/criterion (see flowcharts suppl. tables 4a and 4b for rating process).

ND = not delivered (*full downgrade*).

NM = indicator was not monitored throughout the study period (*full downgrade*).

* = indicator was not monitored at T6 (one *downgrade* on criterion 1).

** = the dataset provided by the institution (T1) contained technical errors. The institution could not properly extract the requested data due to unforeseen issues with its new data warehouse system. *To minimize possible bias*: erroneous data

(on ***registration contact persons/waiting lists/staff turnover***) was not included in the rating procedure for step 2 (criteria 1, 2, and 3) and was also removed from further analyses. Data provided by the institution on ***safety plans*** (T1) was

deemed valid and therefore included in the rating procedure.

*** = SUPRANET’s threshold values for safety plans, staff turnover, involvement of families/caretakers, and waiting lists (*int period*).

**** = the institution had a borderland score on subscale 3.

^1^ = data collection of the indicator started at T1.

^a^ = institution did not monitor indicator (safety plans) at T0, T1 and T2 (one *downgrade* on criterion 1).

^b^ = institution did not monitor indicator (safety plans) at T0 (one *downgrade* on criterion 1).

^c^ = institution did not monitor indicator (registration contact persons) at T1 and T2 (one *downgrade* on criterion 1).

^d^ = institution did not monitor indicator (registration contact persons) at T2 (one *downgrade* on criterion 1).

^e^= institution did not monitor indicator (waiting list duration) at T0 (one *downgrade* on criterion 1).

^f^ = institution incorrectly monitored indicator (staff turnover) at T3 (one *downgrade* on criterion 1).
