# Supplementary material for: Reducing suicides in mental healthcare: results from a 4-year follow-up implementation study in the Netherlands (SUPRANET)
Source: Front Psychiatry. 2024 Apr 19;15:1080235. doi: 10.3389/fpsyt.2024.1080235 (PMC11068092; doi:10.3389/fpsyt.2024.1080235)
Supplement: Supplementary file 1 [file DataSheet_1.zip › Supplementary Tables and Figures.docx]

**Supplementary Table 1.** Minimal Dataset

For quality indicators measured categorically such as safety plans, the response category ‘*1=yes*’ was used to indicate how many patients received a safety plan during treatment. The same was done for registration of contact persons (*1=yes*) and waiting list duration (*1= <2 weeks*). Staff turnover was collected as a continuous variable (percentages). Throughout the total study period, the Minimal Dataset (MDS) underwent several changes (in terms of variables and categories).

Below a description of how each quality indicator was defined and operationalized:

1. Safety plan(s): *A patient has a crisis plan, safety plan, or alert plan (not older than one year) during the data collection period* (between [DD-MM-YYYY] and [DD-MM-YYYY])*. This period was measured as the time between the date the safety plan was created/updated and the treatment end date (discharged/end of treatment trajectory), or if treatment was still ongoing, the end date of the data file* (0= no, 1= yes or unknown/other category). The no category (= 0) also applied for safety plans older than one year.
2. Waiting list duration: *The period between a patient’s referral to a mental health institution (T0) and first treatment contact (T2) (as determined by the Dutch Healthcare Authority (NZa) in The Netherlands)* (1= <2 weeks, 2= > 2 weeks to < 4 weeks, 3= >4 weeks or unknown/other category).
3. Registration of a contact person (involvement of families/caretakers): *At least one contact person (name and telephone number of family member) was registered in the patient’s health record (EHR) such as name and phone number of family member(s)/carers/friends*) (0= no, 1= yes or unknown/other category).
4. Staff turnover (measured as a continuous variable (in percentages)): *The number of employees on [DD-MM-YYYY] plus the number of employees on [DD-MM-YYYY], divided by two (without taking into account freelancers). This results in the average number of employees during that time period. Then divide the number of employees leaving an institution during the data collection period [DD-MM-YYYY] and [DD-MM-YYYY] by the average number of employees.*

Example of the Minimal Dataset (last data collection period; (T8)).

| **Name + type variable** | **Specification / categories**  (categories are mutually exclusive) | **Definition** |
| --- | --- | --- |
| *Year*  Numeric | Data collection period: first or second half of the year (e.g. [YYYY]_1 or [YYYY]_2) | Year of the delivery file. Including  whether it concerns the first or second  half of the year (e.g., [YYYY]_1 or  [YYYY]_2). |
| *Gender_cat*  Numeric | - Unknown - Male - Female - Other | Gender category in text. |
| *Age_cat*  Numeric | - 0 – 17 years - 18 – 39 years - 40 – 59 years - 60 – 79 years - >= 80 years - Unknown | Age in categories.  Age is calculated by year of the delivery file minus the birth year. |
| *Setting*  Numeric | - General practice-based (nurse) specialist in primary care (POH-GGZ) - General basic mental healthcare (GB-GGZ) - Youth mental healthcare (Jeugdhulp) - Specialist mental healthcare and/or forensic care (S-GGZ / FZ-GGZ) - Long-term care (WLZ / L-GGZ) - Unknown - Other | The setting only relates to the care that was provided during the data collection period covered by the delivery file.  *If a patient received both specialist mental healthcare (S-GGZ) and general basic mental healthcare (GB-GGZ) within the same time period, we chose the most severe setting (in this case S-GGZ). The categories S-GGZ and FZ-GGZ (i.e., forensic care) have been put in equal footing in terms of severity.* |
| *Admission* (previous variable: type of care; e.g., inpatient care/outpatient care)^3^  Numeric | - None - < 28 days - > 28 days | Number of days a patient was admitted during the (data collection) period covered by the delivery file. |
| *Marital_status*  Numeric | - Not married - Married/ registered partnership - Divorced - Widowed - Unknown - Other | Marital status at last registration. |
| *Psych_DSMdiagnosis*^1^  Numeric | - Alcohol-related disorders - Other conditions that may be a focus of clinical attention - Anxiety disorders - Medication induced movement disorders and other adverse effects of medication - Bipolar- and other mood disorders - Depressive disorders - Disruptive, impulse-control, and conduct disorders - Dissociative disorders - Gender dysphoria - Neurodevelopmental disorders - Neurocognitive disorders - Obsessive compulsive and related disorders - Other substance use disorders - Paraphilic disorders - Personality disorders - Schizophrenia and other psychotic disorders - Sexual dysfunctions - Sleep-wake disorders - Somatic symptom and related disorders - Elimination disorders - Trauma- and stressor-related disorders - Feeding and eating disorders - Unknown - Other mental disorders (adults) - Other (e.g., *youth care*) | Most recent DSM-5 (main)-diagnosis.  For youth up to 17 years: fill in where possible, otherwise use ‘other’ option. |
| *Safety plan*  Numeric | - No - Yes - Unknown | Patient has a safety plan/ crisis plan (not older than 1 year) during the period covered by the delivery file.  This period was measured as the time between the date the safety plan was created/updated and treatment end date (discharged/ end of treatment trajectory), or if treatment is still ongoing, the end date of the datafile.  *For safety plans older than 1 year, the ‘’no’’-category should be selected.* |
| *Waitinglist_duration*  Numeric | - < 2 weeks - >= 2 weeks to =< 4 weeks - > 4 weeks - Unknown | Waiting list duration is the period between a patients’ referral to a mental health institution (T0), and his/her first treatment contact (T2). |

| *Recently_in_care*  Numeric | - No - Yes - Unknown | Patients had their first treatment contact (T2) at some point during the data collection period. |
| --- | --- | --- |
| *Registr_ contactperson*  Numeric | - No - Yes - Unknown | For each patient, at least one contact person is registered in the EHR^2^ (name and phone number of family member(s)/carers/friends/other). |
| *Treatment_duration*  Numeric | - < 3 months - >= 3 months to =< 1 year - > 1 year - Unknown | Treatment duration is the period between a patient’s first treatment contact (T2) and treatment end date OR the end date of the data file (*in case therapy trajectory is still ongoing*).  For patients who have finished or stopped their treatment, this variable is computed as follows: treatment end date minus date of first treatment contact.  For patients who are still admitted/enrolled into treatment: the end date of the datafile [DD-MM-YYYY] minus the date of first treatment contact. |
| *Number_clients*  Numeric |  | The number of unique patient identifiers having an active record in the EHR^2^ during the data collection period. |
| *Number_suicides*  Numeric |  | This is the number of suicides [1] as determined by the medical director or by the board of directors (in Dutch: RvB) of the mental health institution. This includes counting suicides of (A) patients who were registered/admitted for treatment at the time of death (by suicide), as well as patients (B) who died by suicide within 14 days of discharge from the institution and who did not (yet) receive care from another (private) practice or mental health institution. |
| *Number_suicide_attempters*  Numeric |  | The number of suicide attempters. A suicide attempter is a patient who has made a suicide attempt within the data collection period. |

^1^ Institutions delivering DSM-5 categories from T6 (second half of 2019) onwards, were recoded into DSM-IV categories for the statistical analyses.

^2^ EHR = electronic health record.

^3^ Adjustments to the variable have been made from T7 onwards (first half of 2020).

**Organizational characteristics**

| **Name variable** | **Definition** |
| --- | --- |
| *Staff turnover*  (in percentages) | Staff turnover between [DD-MM-YYYY] and [DD-MM-YYYY]. Calculation = (number of employees on [DD-MM-YYYY] + the number of employees on [DD-MM-YYYY] divided by 2, without taking into account freelancers. This is the average number of employees during that period. Next, divide the number of employees who quit working at the institution between [DD-MM-YYYY] and [DD-MM-YYYY] by the average number of employees. |

**Supplementary Table 2.**

The self-report questionnaire was filled out by the institutions and consists of four subscales. The following three subscales measured the distribution and usage of the SUPRANET intervention: 1) how many participants from an institution have read the *last* feedback report or *any* feedback report (*measured as: yes/no*), 2) if the institutions distributed the biannual feedback reports* organization-wide (*five-point Likert scale*), and 3) if institutions formulated and monitored best practices related to safety plans, involvement of family and caretakers, waiting lists, and staff turnover (*measured on a five-point Likert scale*)). The fourth subscale (*content of feedback reports**) measured a different construct and was therefore excluded from the analysis.

Total scores (SD) for the arrival of the SUPRANET intervention within the participating institutions (*N*=29)

| **Questionnaire** | **Item** | **Aspects** | | **Groups of participants** | | | |  |
| --- | --- | --- | --- | --- | --- | --- | --- | --- |
|  |  |  | | Directors, medical directors, and board members (*n*=15) | Chairpersons of suicide prevention board (*n*=6) | Chairpersons of patients’ and families’ advisory boards (*n*=8) | Total (*n*=29) |  |
| **Subscales** |  |  | | **N (%)** | | | |  |
| **I** |  | **Have read the *last* feedback report or *any* feedback report (*dichotomous score*)?*** |  |  |  |  |  |  |
|  |  |  | |  |  |  |  |  |
|  | 1. | Did you read the most recent (last) biannual SUPRANET Care feedback report (period: July to December 2017? | Yes | 11 (73.3) | 6 (100) | 3 (37.5) | 20 (69.0) |  |
|  |  |  | No | 4 (26.7) | 0 (0.0) | 5 (62.5) | 9 (31.0) |  |
|  | *If participants answered ‘no’ on the first item:* | | |  |  |  |  |  |
|  | 2. | Did you read one of the other SUPRANET Care feedback reports (i.e., first or second published report? | Yes | 1 (6.7) | 0 (0.0) | 1 (12.5) | 2 (6.9) |  |
|  |  |  | No | 3 (20.0) | 0 (0.0) | 4 (50.0) | 7 (24.1) |  |
|  |  |  | NA^1^ | 11 (73.3) | 6 (100) | 3 (37.5) | 20 (69.0) |  |
|  |  |  | | **Total scores (*SD*)** | | | | **Range** |
| **II** |  | **Distribution and usage of feedback reports^2.*^** |  | **36.00 (6.25)** | **26.67 (9.67)** | **39.67 (12.09)^3^** | **33.63 (9.33)** | **10 - 50** |
|  |  |  | |  | | | |  |
|  |  |  | | **Item scores (*SD*)** | | | | **Range** |
|  | 1. | Feedback reports are discussed during meetings |  | 3.45 (0.52) | 2.83 (0.75) | 3.25 (1.50) | 3.24 (0.83) | 1 - 5 |
|  | 2. | Feedback reports are discussed with colleagues |  | 3.92 (1.17) | 2.33 (0.52) | 4.50 (0.58) | 3.59 (1.22) |  |
|  | 3. | Colleagues stimulate each other to read the feedback reports |  | 3.58 (1.17) | 2.33 (1.03) | 4.50 (0.58) | 3.41 (1.26) |  |
|  | 4. | Medical directors have read the feedback reports |  | 4.91 (0.30) | 4.75 (0.50) | 4.50 (1.0) | 4.79 (0.54) |  |
|  | 5. | Board members have read the feedback reports |  | 4.17 (1.12) | 3.25 (1.71) | 4.33 (1.16) | 4.0 (1.25) |  |
|  | 6. | Suicide prevention committee members have read the feedback reports |  | 4.83 (0.39) | 4.17 (1.60) | 4.25 (0.96) | 4.55 (0.96) |  |
|  | 7. | Team managers have read the feedback reports |  | 4.25 (0.71) | 2.40 (1.52) | 4.50 (0.71) | 3.67 (1.35) |  |
|  | 8. | Feedback reports are actively distributed in newsletters or intranet (web portal for employers) |  | 2.60 (1.58) | 1.60 (0.89) | 3.0 (1.83) | 2.42 (1.50) |  |
|  | 9. | Employees are informed about the existence of the feedback reports |  | 2.80 (1.32) | 2.17 (1.17) | 3.67 (2.31) | 2.74 (1.45) |  |
|  | 10. | Employees know how to access the feedback reports |  | 2.40 (1.27) | 1.60 (0.89) | 3.0 (2.83) | 2.24 (1.35) |  |
|  |  |  | | **Total scores (*SD*)** | | | | **Range** |
| **III** |  | **Formulating best practices^4,*^** |  | **34.61 (3.88)** | **31.02 (6.74)** | **38.0 (1.41)^5^** | **33.87 (5.05)** | **9 - 45** |
|  |  |  | | **Item scores (*SD*)** | | | | **Range** |
|  | 1. | The feedback reports are used to change existing suicide prevention policies within the institutions |  | 3.50 (0.91) | 3.0 (1.09) | 3.75 (1.26) | 3.41 (1.01) | 1 – 5 |
|  | 2. | Institutions formulated one or more best practices on the theme ‘*involving of family or significant others*’ |  | 3.90 (0.74) | 2.60 (1.34) | 4.0 (1.0) | 3.56 (1.10) |  |
|  | 3. | Institutions formulated one or more best practices on the theme ‘*safety plans’* |  | 4.10 (0.74) | 3.50 (1.52) | 4.0 (1.41) | 3.89 (1.08) |  |
|  | 4. | Core teams monitor best practices within the institutions |  | 4.33 (0.89) | 3.67 (0.82) | 4.75 (0.50) | 4.23 (0.87) |  |
|  | 5. | (Medical) directors are informed about the results of best practices (e.g., *staff turnover, waiting lists*) |  | 4.08 (0.99) | 3.75 (1.50) | 5.0 (0.0) | 4.11 (1.08) |  |
|  | 6. | Institutions learn from best practices by sharing their results on the SUPRANET website/ new letters and/or during annual exchange meetings |  | 3.25 (0.97) | 3.25 (0.96) | 4.0 (1.41) | 3.33 (0.80) |  |
|  | *If applicable (optional items):* | | |  |  |  |  |  |
|  | 7. | Institutions have changed their existing suicide prevention policies after reading the feedback reports |  | 3.27 (0.65) | 3.17 (1.17) | 3.67 (0.58) | 3.30 (0.80) | 1 - 5 |
|  | 8. | Institutions are confident about their own suicide prevention policies after reading the feedback reports |  | 3.91 (0.70) | 3.50 (0.58) | 3.67 (0.58) | 3.78 (0.65) |  |
|  | 9. | Institutions  are sure to continue with their own suicide prevention policies after reading the feedback reports |  | 4.50 (0.52) | 4.50 (0.55) | 4.50 (0.71) | 4.50 (0.51) |  |

SD = standard deviation.

* = The items of subscales I, II and III are about the third biannual feedback report (period: July to December 2017) and the (two) feedback reports published before that.

^1^ NA= not applicable.

^2^ Total score contains ten missing values after using the person mean substitution method (subscale II; distribution and usage of feedback reports).

^3^ Three observations included in this group of participants.

^4^ Total score contains nine missing values after using the person mean substitution method (subscale III; formulating best practices).

^5^ Two observations are included in this group of participants.

**Supplementary Table 3A.** Rating process to classify institutions for ***step 1*** (distribution and usage of the SUPRANET intervention)

a

|  |
| --- |

**Supplementary Table 4b.** Decision-making process to classify institutions for step 2 (compliance on SUPRANET quality indicators).

**Subscale 3:**

**Were best practices formulated?**

*(no downgrade or one downgrade)*

*(no downgrade or full downgrade)*

**Subscale 2:**

**Were the feedback reports adequately distributed?**

*(no downgrade or full downgrade)*

**Subscale 1:**

**How many participants have read *any* or *last* feedback report?*****

*(no downgrade, one downgrade or full downgrade)*

**Rating**

**Classification**

No / MD*

***ABOVE AVERAGE:***

**OR**

|  |
| --- |

Cut-off > **26.5**

|  |
| --- |

All participants (***100%***)

Cut-off > **32**

Yes

Yes

|  |
| --- |

|  |
| --- |

No / MD

Most participants *(****>50% to <100%****)*

Yes

Yes

*ORm*

No / MD

No / MD*

Half (or less) of the participants *(****<50%****)** OR* institution delivered no participants to fill out the survey (***ND***)

Institution not assessed on subscale(s) *, **

.

***** If feedback reports were not distributed and used as intended (subscale 2), it served as a sign that the SUPRANET intervention was not properly executed (*institution received a full downgrade for step 1*).

******If half (or less than half) of the participants have read the feedback report (subscale 1), it served as a sign that the SUPRANET intervention was not executed as intended (*institution received a full downgrade for step 1*).

***The items of subscale I (*Supplementary Table 2*) are about the third feedback report (i.e., *last feedback report*, period: *July to December 2017*) and the (two) feedback reports published before that.

MD = missing data (cannot be assessed).

ND = institution delivered no participants to fill out the survey.

**Supplementary Table 3B.** Rating process to classify institutions for ***step 2*** (compliance with the SUPRANET quality indicators)

|  |
| --- |

**Supplementary Table 4b.** Decision-making process to classify institutions for step 2 (compliance on SUPRANET quality indicators).

***Criterion 1:***

**Is the indicator monitored on *every biannual time point*?**

*(no downgrade, one downgrade, or full downgrade)*

***Criterion 3 (optional):***

**Was there an improvement in monitoring the indicator *over time* (*baseline vs intervention*)?**

*(no downgrade, or one downgrade)*

**Improvement of indicator at baseline vs intervention (criterion 1B)**

**Improvement of indicator at baseline vs intervention (criterion 1B)**

**Improvement of indicator at baseline vs intervention (criterion 1B)**

***Criterion 2:***

**Was the indicator monitored better than the total network (*during the intervention period*)?**

*(no downgrade or one downgrade)*

**Level of compliance towards the indicator compared to SUPRANET average (criterion 1A)**

**Classification**

**Rating**

**Indicator**

***Indicators***

|  |
| --- |

***ABOVE AVERAGE:***

**At least 2 indicators:**

**AND**

**At least 1 indicator:**

|  |
| --- |

Yes*

Monitored on *every biannual timepoint (****Yes****)*

No

Yes

Improved?

> **16.6%**

|  |
| --- |

No/MD

1. Safety plans

No

Yes/No/ MD

Not monitored *on every timepoint (****No****)*

|  |
| --- |

Yes*

*Not monitored at all (****NM****)***

|  |
| --- |

Yes*

Improved?

> **43.4%**

Monitored on *every biannual timepoint (****Yes****)*

|  |
| --- |

No

Yes

|  |
| --- |

No/MD

Not monitored *on every timepoint (****No****)*

2. Registration contact person

No

Yes*

Yes/No/ MD

|  |
| --- |

|  |
| --- |

*Not monitored at all (****NM****)***

|  |
| --- |

|  |
| --- |

Yes*

Yes

Improved?

Monitored on *every biannual timepoint (****Yes****)*

> **20.1%**

No

No/MD

3. Waiting list duration

|  |
| --- |

No

Yes/No/ MD

Not monitored *on every timepoint (****No****)*

*ORm*

Yes*

|  |
| --- |

|  |
| --- |

*Not monitored at all (****NM****)***

|  |
| --- |

Yes*

Improved?

< **9.9%**

Monitored on *every biannual timepoint (****Yes****)*

|  |
| --- |

No

Yes

No

Yes*

No/MD

Yes/No/ MD

*Not monitored at all (****NM******)*

Not monitored *on every timepoint (****No****)*

MD = missing data

Institution not assessed on criterion

4. Staff turnover

*****If institutions adequately monitored an indicator during the intervention period (criterion 2), they were not assessed on criterion 3 (*did the institution make any efforts better monitor the indicator over time (baseline vs intervention period*)), as they already monitored the indicator better than average.

******If an indicator was not monitored at all (criterion 1; ***NM***), it served as a sign that the institution did not register the indicator(s) as intended, nor were any efforts made to improve its registration (*the institution received a full downgrade on the indicator: safety plans/ staff turnover/ registration contact persons/ waiting lists* (step 2)).

MD = missing data (cannot be assessed).

**Supplementary Table 5.** Characteristics of all patients in care (in percentages) within the 13 institutions

at baseline (T0-T1: institutional level (Figure 1))

| Characteristics | All patients in care (%) |
| --- | --- |
| % Gender |  |
| Male | 49.5 |
| Female | 50.5 |
| % Age |  |
| 0 – 19 years | 15.0 |
| 20 – 39 years | 34.8 |
| 40 – 59 years | 33.6 |
| 60 – 79 years | 13.1 |
| > 80 years | 3.5 |
| % Marital status |  |
| Unknown/other | 32.4 |
| Not married | 42.5 |
| Married | 16.8 |
| Divorced | 6.5 |
| Widowed | 1.8 |
| % Setting |  |
| Unknown/other | 2.6 |
| General practice-based (nurse) specialist in primary care (POH-GGZ) | 0.3 |
| General basic mental healthcare (GB-GGZ) | 10.0 |
| Long-term care (WLZ) | 2.3 |
| Specialist healthcare (S-GGZ) or forensic care (FZ-GGZ) | 84.8 |
| % Main categories DSM-IV diagnosis |  |
| Unknown | 13.0 |
| Substance use disorders (alcohol disorders and other  substance use disorders)^a^ | 7.1 |
| Adjustment disorders | 0.2 |
| Attention-deficit and disruptive behavior disorders | 6.5 |
| Other conditions that may be a focus of clinical attention | 0.4 |
| Anxiety disorders | 13.0 |
| Delirium, dementia and other cognitive disorders | 3.0 |
| Mood disorders (depression, bipolar and (other)  mood disorders)^b^ | 19.7 |
| Eating disorders | 1.1 |
| Other developmental disorders | 3.4 |
| Personality disorders | 8.8 |
| Pervasive development disorders | 5.5 |
| Not otherwise specified | 4.1 |
| Schizophrenia and other psychotic disorders | 10.8 |
| Somatoform disorders | 1.2 |
| Other | 2.2 |
| % Treatment duration |  |
| Unknown/other | 15.4 |
| < 3 months | 16.1 |
| > 3 months and < 1 year | 26.5 |
| > 1 year | 42.0 |
| % Type of care |  |
| Unknown/other | 1.5 |
| Outpatient care | 90.1 |
| Inpatient care | 8.4 |

^a^ Alcohol disorders and other substance use disorders were merged as one category.

^b^ Depressive disorders, bipolar and other mood disorders were merged as one category.

**Supplementary Table 6.** Characteristics (in percentages) of mental health professionals (*N*=400) working in ambulatory care teams and crisis teams (from 12 mental health institutions) at baseline (T0: team level (Figure 1))

| Characteristics | Crisis teams  (*n*=246) | Ambulatory care teams (*n*=154) | Total  (*N*=400) |
| --- | --- | --- | --- |
| % Working experience | | | |
| < 1 year | 5.3 | 6.5 | 5.8 |
| 1 – 2 years | 4.1 | 3.9 | 4.0 |
| 3 – 5 years | 8.9 | 9.1 | 9.0 |
| 6 – 10 years | 14.2 | 12.3 | 13.5 |
| > 10 years | 67.5 | 68.2 | 67.8 |
| Mean number of patients with suicidality treated during the past four weeks (M; SD) | 15.35 (13.1) | 9.03 (9.37) | 12.92 (12.21) |
| % Profession |  |  |  |
| Nurse | 22.8 | 9.7 | 17.8 |
| Social psychiatric nurse (SPV) | 31.7 | 17.5 | 26.3 |
| Social worker | 1.6 | 0.6 | 1.3 |
| Creative therapist | 0.0 | 3.9 | 1.5 |
| Nurse practitioner | 2.4 | 3.9 | 3.0 |
| Psychologist or education generalist | 0.0 | 5.2 | 2.0 |
| Psychologist in training to become  a health psychologist | 0.0 | 6.5 | 2.5 |
| Licensed health psychologist | 1.6 | 14.3 | 6.5 |
| Licensed health psychologist in training  to become a clinical psychologist | 0.4 | 4.5 | 2.0 |
| Clinical psychologist | 0.8 | 7.1 | 3.3 |
| Psychotherapist | 0.0 | 1.3 | 0.5 |
| Specialist trainee in psychiatry | 12.2 | 5.8 | 9.8 |
| Psychiatrist | 19.9 | 14.9 | 18.0 |
| Other | 6.5 | 4.5 | 5.8 |
| % Training total^a^ |  |  |  |
| Training: Yes, participated in a training | 79.7 | 66.9 | 74.8 |
| Training: No, never participated in a training | 19.5 | 33.1 | 24.8 |
| % Training^a^ |  |  |  |
| PITSTOP – training: Yes | 57.3 | 50.0 | 54.5 |
| PITSTOP – training: No | 41.9 | 50.0 | 45.0 |
| Other suicide prevention training: Yes | 65.4 | 46.8 | 58.3 |
| Other suicide prevention training: No | 33.7 | 53.2 | 41.3 |

*M:* mean, *N*: sample size; *SD*: standard deviation.

^a^Two missing values (within ‘crisisteam’ category) for PITSTOP training and other suicide prevention training.


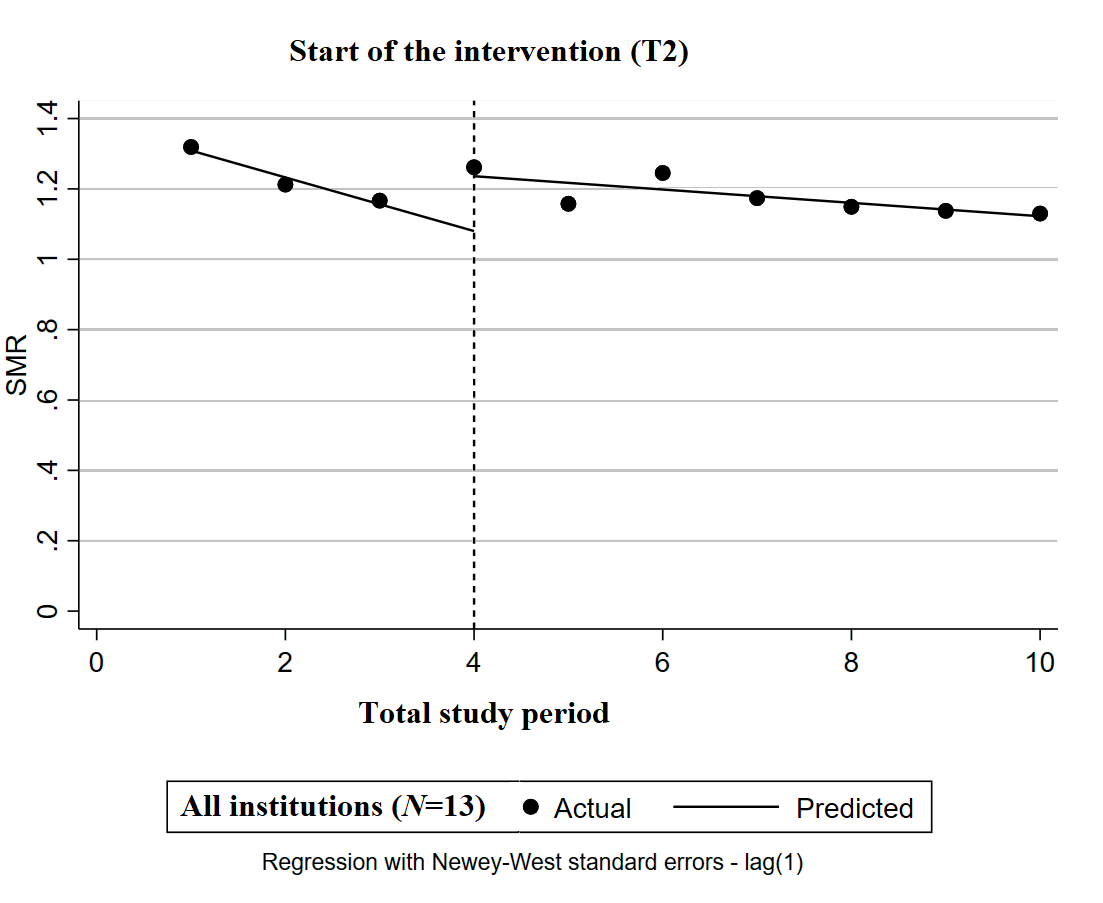
**Supplementary Figure 1**. Single-group ITSA on suicides (measured as: standardized mortality ratio (SMR)) with Newey-West standard errors and one lag (total sample (*N*=13))

**Supplementary Figure 2.** Single-group ITSA for proportion of patients with reported suicide attempts with Newey-West standard errors and two lags (total sample (*N*=13))


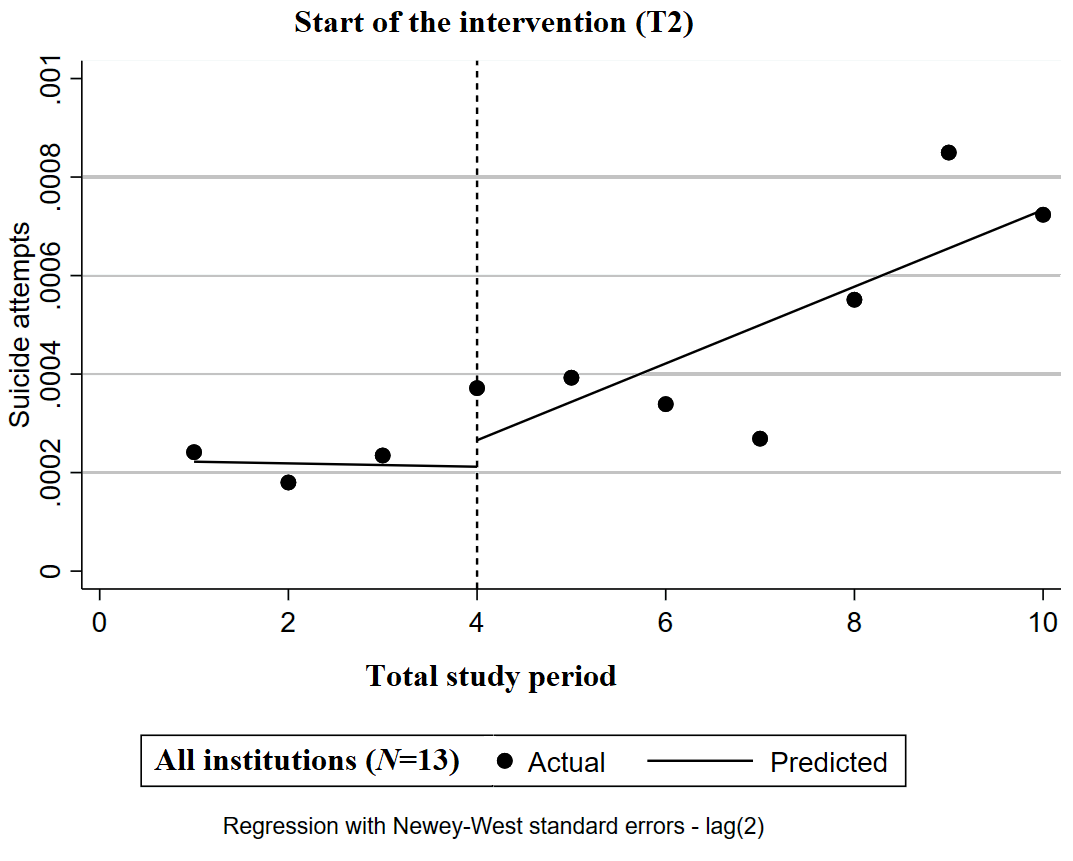


**Supplementary Figure 3.** Longitudinal changes in professionals’ knowledge between institutions labeled as ***above average*** (complied with the quality indicators and performed the SUPRANET intervention more as intended) and institutions labeled as ***below average*** (adjusted model)

**
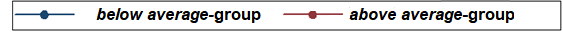

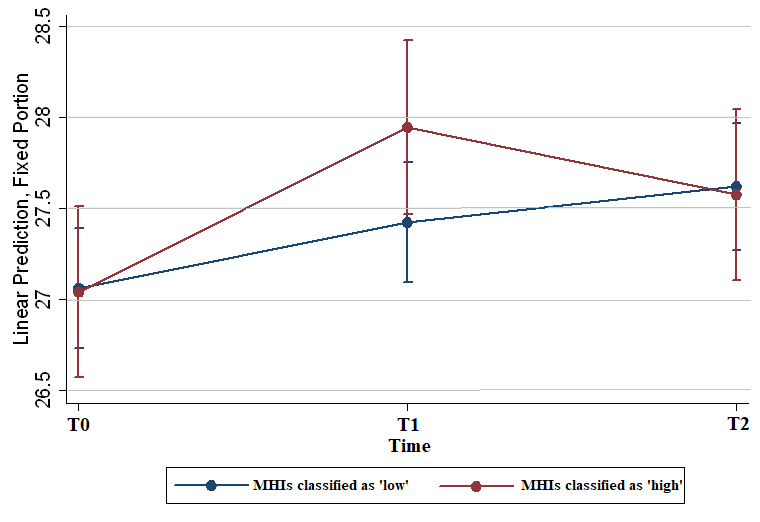
**

**Supplementary Figure 4.** Longitudinal changes in professionals’ attitude between institutions labeled ***as above average*** (complied with the quality indicators and performed the SUPRANET intervention more as intended) and institutions labeled as ***below average*** (adjusted model)

**
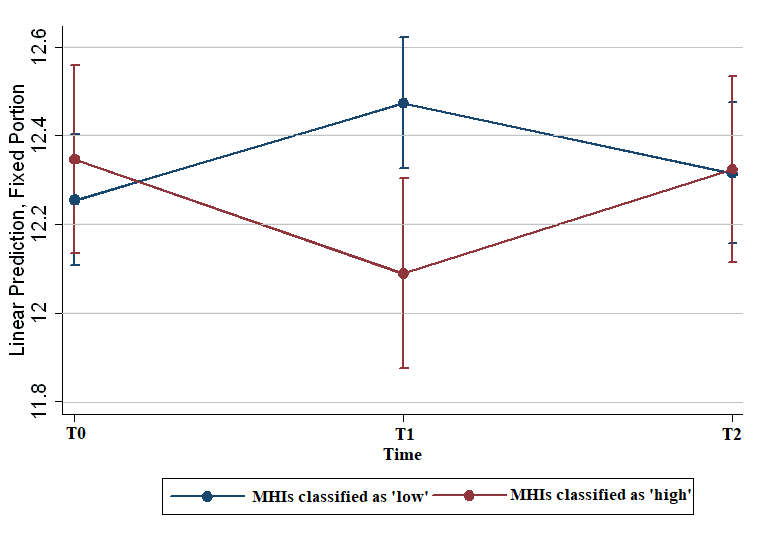
**

**
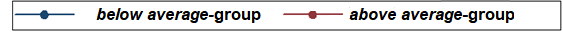
**

**Supplementary Figure 5.** Longitudinal changes in professionals’ adherence to the guideline between institutions labeled as ***above average*** (complied with the quality indicators and performed the SUPRANET intervention more as intended) and institutions labeled as ***below average*** (adjusted model)


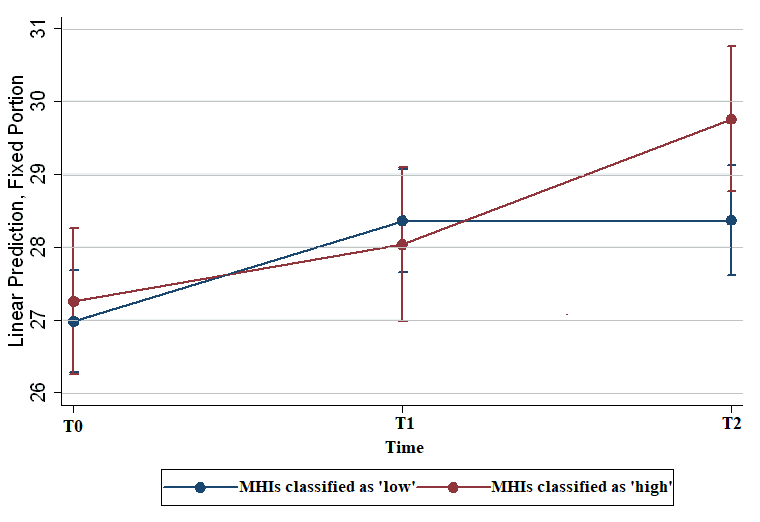


**
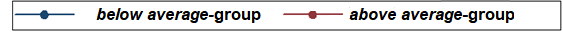
**
